# Attention for future reward

**DOI:** 10.1007/s00426-018-1094-4

**Published:** 2018-09-11

**Authors:** Helen Tibboel, Baptist Liefooghe

**Affiliations:** 1 Erasmus School of Social and Behavioural Sciences, Erasmus University, Burgemeester Oudlaan 50, 3062 PA Rotterdam, The Netherlands; 2 Department of Experimental-Clinical and Health Psychology, Ghent University, Ghent, Belgium

## Abstract

When stimuli are consistently paired with reward, attention toward these stimuli becomes biased (e.g., Abrahamse, Braem, Notebaert & Verguts, et al., Psychological Bulletin 142:693–728, 2016, 10.1037/bul0000047). An important premise is that participants need to repeatedly experience stimulus–reward pairings to obtain these effects (e.g., Awh, Belopolsky & Theeuwes, Trends in Cognitive Sciences 16:437–443, 2012, 10.1016/j.tics.2012.06.010). This idea is based on associative learning theories (e.g., Pearce & Bouton, Annual Review of Psychology 52:111–139, 2001) that suggest that exposure to stimulus–reward pairings leads to the formation of stimulus–reward associations, and a transfer of salience of the reward to the neutral stimulus. However, novel learning theories (e.g., De Houwer, Learning and Motivation 53:7–23, 2009, 10.1016/j.lmot.2015.11.001) suggest such effects are not necessarily the result of associative learning, but can be caused by complex knowledge and expectancies as well. In the current experiment, we first instructed participants that a correct response to one centrally presented stimulus would be followed by a high reward, whereas a correct response to another centrally presented stimulus would be paired with a low reward. Before participants executed this task, they performed a visual probe task in which these stimuli were presented as distractors. We found that attention was drawn automatically toward high-reward stimuli relative to low-reward stimuli. This implies that complex inferences and expectancies can cause automatic attentional bias, challenging associative learning models of attentional control (Abrahamse et al., 2016; Awh et al., 2012).

## Introduction

The last decade has seen a surge in studies on the effects of reward on attentional control (for reviews, see Abrahamse, Braem, Notebaert, & Verguts, 2016; Anderson, 2016; Chelazzi, Perlato, Santandrea, & Della Libera, 2013; Pessoa, 2014). For instance, visual search is more efficient when a reward-related stimulus functions as a target (e.g., Kristjansson et al., 2010) and less efficient when this stimulus functions as a distractor (e.g., Anderson, Laurent & Yantis, 2011; Anderson & Yantis, 2012). Recent reviews distinguish different types of reward effects. There are short-term reward priming effects, in which a stimulus that is associated with reward on trial N-1 grabs attention on trial N, and longer-lasting reward biases that are the result of consistently presenting participants with stimulus–reward pairings in an extensive training phase (Pessoa, 2014). The latter effects last over large numbers of trials and have even been reported up to 6 months after training (Anderson & Yantis, 2013; also see Anderson et al., 2011; Della Libera & Chelazzi, 2009). Furthermore, they occur even when rewards are no longer given (e.g., Anderson et al., 2011; Lee & Shomstein, 2014) and when attending to reward-related stimuli is detrimental for the task at hand (e.g., Le Pelley, Pearson, Griffiths, & Beesley, 2015; Pearson, Donkin, Tran, Most, & Le Pelley, 2015).

Until now, the general assumption regarding reactive effects of reward on attention is that they can be achieved only through associative learning (e.g., Abrahamse et al., 2016) and require a learning history of at least hundreds of trials (e.g., Pessoa, 2014). It is proposed that the repeated paring of a stimulus and a reward leads to the formation of associations between the representation of the stimulus (commonly referred to as the conditioned stimulus or CS) and the representation of the reward (commonly referred to as the unconditioned stimulus or US; e.g., Anderson, 2016). Once this association has been formed, the salience and automatic “attention-grabbing” properties of the reward are assumed to transfer to the neutral stimulus, resulting in an attentional bias toward the CS (for a review on associative learning, see Pearce & Bouton, 2001).

On the basis of this idea, a new model of attentional processing has been proposed by Awh et al. (2012; see also Failing & Theeuwes, 2018; Theeuwes, 2018), who suggest that the classical dichotomy of top-down and bottom-up attentional selection does not hold. Instead, they introduced two novel modes of attentional control: selection and reward history. When participants have a history of attending toward specific stimuli (e.g., because doing so results in receiving a high reward), they develop an attentional bias toward these stimuli. These effects are automatic in the sense that they are not strategic or top-down, because they still occur when rewards are no longer presented, or when attending to reward-related stimuli is detrimental for performance (e.g., Anderson et al., 2011); neither are they bottom-up: the stimuli do not have visual features that make them “pop-out” (e.g., Theeuwes, 1991). Instead, they suggest that the attentional bias toward reward cues can be explained only by participants’ experience with the stimulus–reward pairings (i.e., reward history).

The perseverance of the idea that automatic attentional bias toward rewarding stimuli is due to a learning history is surprising, as developments within learning psychology have led to novel theories that suggest that it is not necessary to train the stimulus–reward pairings for hundreds of trials to obtain automatic effects. Instead, instructions regarding future (e.g., not experienced) events can also lead to the formation of automatic associations in memory (e.g., Gawronski & Bodenhausen, 2011; Ramamoorthy & Verguts, 2012). Other, propositional, learning theories go one step further and propose that the formation of associations is unnecessary to obtain automatic effects. Instead, automatic effects can be due to complex knowledge (De Houwer, 2009; 2015; Mitchell, De Houwer, & Lovibond, 2009). On the basis of these theories, it is expected that instructions regarding stimulus–reward pairings should also affect automatic attention.

Research has indeed shown that mere instructions have powerful effects on automatic behavior. For instance, instructions regarding the future pairing between a CS and an evaluative US affects the automatic evaluation of the CS, even though these pairings were not experienced (e.g., Mertens, Raes, & De Houwer, 2016). Similarly, Van Dessel, De Houwer, & Gast (2015) showed that instructing participants to approach or avoid a specific CS affected participants’ automatic evaluation of this CS. Instructions also have automatic effects in non-affective domains. For instance, Liefooghe, Wenke, and De Houwer (2012) showed that instructed stimulus–response (S-R) mappings can result in the automatic activation of responses and influence behavior on other tasks (see also Everaert, Theeuwes, Liefooghe, & De Houwer, 2014; Wenke, De Houwer, De Winne, & Liefooghe, 2015; see Brass, Liefooghe, Braem, & De Houwer, 2017; Meiran, Liefooghe, & De Houwer, 2015 for reviews). Furthermore, previous research has shown that merely holding items in working memory can bias attention toward these items (for a review, see Olivers, Peters, Houtkamp, & Roelfsema, 2011). Reactions are faster when targets are presented at the same location as a to-be-remembered stimulus (e.g., Dowd & Mitroff, 2013; Downing, 2000; Schwark, Dolgov, Sandry, & Volkman, 2013; Soto, Humphreys, & Heinke, 2008) and slower when it is presented at another location from a to-be-remembered stimulus (e.g., Soto & Humphreys, 2007, 2008). Finally, Tibboel, Liefooghe, and De Houwer (2016) gave participants instructions regarding future S–R mappings and examined attentional bias toward these stimuli. They reported an attentional bias toward stimuli that were linked to a future response compared to control stimuli. Interestingly, Tibboel et al. failed to find similar effects on the basis of instructed S–S mappings. This suggests that, whereas working memory alone can bias attention, it does not do so unconditionally.

**Fig. 1:**
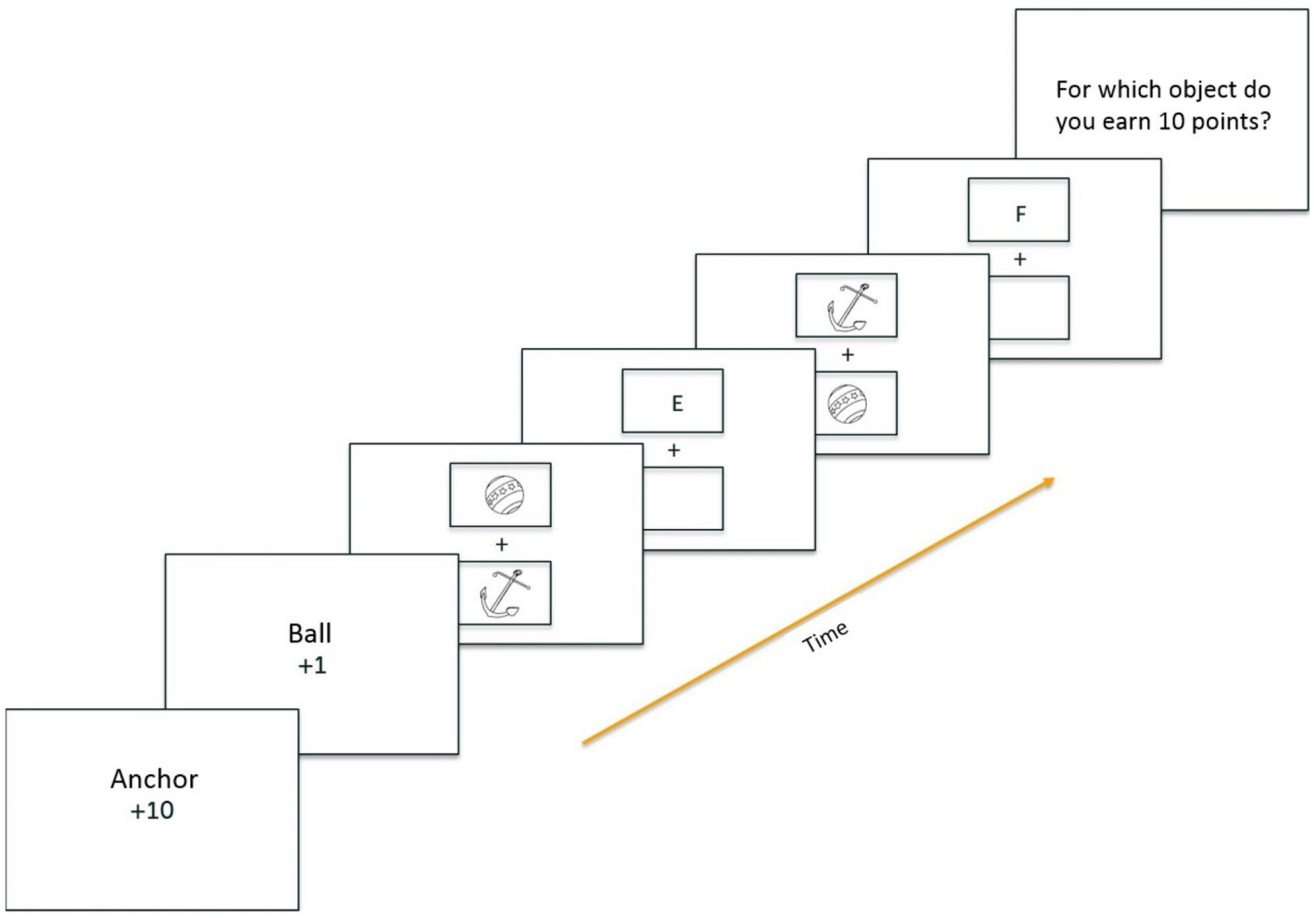
Schematic example of a run in experiment 1

The aim of the current paper is to examine the effects of instructed reward on attention. To examine this question, we adapted the procedure of Tibboel et al. (2016). Our experiment consisted of two tasks, an inducer task and a diagnostic task. First, participants received instructions for the inducer task: there were two object names that were associated with either a high (10 points) or low (1 point) reward (see Fig. 1 for an example). Participants were told that at an arbitrary moment, one of two following questions would appear in the center of the computer screen: “For which object do you earn 1 point?” or “For which object do you earn 10 points?” If participants answered this question correctly, they would earn the corresponding number of points. Participants who earned the most points were entered in a lottery for a gift certificate. Thus to win the gift certificate, participants were required to memorize both the high- and the low-reward stimulus.

After participants received the instructions for the inducer task, but before they were required to execute the task, they performed the diagnostic task. This was a visual probe task that required the identification of a probe (the letter E or F) and was designed to measure automatic attentional bias (e.g., Tibboel et al., 2016). Importantly, the visual probe was preceded by the presentation of pictures of the two instructed objects from the inducer task. There were two types of trials: valid trials, in which the probe appeared in the location of the high-reward object, and invalid trials, in which it appeared in the location of the low-reward object. After 8 or 16 visual probe trials, one of the two inducer questions appeared, and when participants answered the question correctly, they received the corresponding number of points (i.e., 1 point for the question “For which object do you earn 1 point?”; 10 points for the question “For which object do you earn 10 points?”). In each run, novel stimuli were used.

Thus, in the current study, we examine whether attention is automatically drawn to high (relative to low) reward stimuli under the following conditions: (1) participants have no previous experience with the stimuli, because the instruction screen always contained words referring to the objects and not the actual images that were used in the visual probe task. This rules out effects of selection history; (2) participants have no previous experience with the stimulus–reward contingencies, because they only receive the reward at the end of each run. This rules out effects of reward history; (3) the high- and low- reward stimuli are not visually salient, excluding effects of bottom-up processes; (4) both high- and low- reward stimuli are irrelevant to the attentional task, ruling out effects of top-down strategies to preferentially attend to the high-reward stimulus; (5) both the high- and low- reward contingencies need to be remembered to perform the inducer task, ruling out effects of strategies to preferentially rehearse the high-reward contingency. Hence, any difference in attention allocation can be due only to the instructed reward value of the stimuli.

Associative learning models predict that this manipulation should not lead to differential allocation of automatic attention, because participants have no training with the stimulus–reward associations. In contrast, the novel learning theories discussed above predict an attentional bias toward high-reward stimuli relative to low-reward stimuli, on the basis of participants’ prior knowledge and expectancies.

## Method

### Participants

Participants were students of Erasmus University Rotterdam. We aimed for a sample of 50 participants, based on power analyses of the data of Experiment 1 of Tibboel et al. (2016), using G-power (Faul, Erdfelder, Buchner, & Lang, 2009). We tested 51 participants, who were rewarded course credit for their participation. Participants who received the most number of points were entered in a lottery to win one €25 gift certificate. There were two versions of the task: one in Dutch and one in English. Forty-one participants were foreign and performed the task in English. Ten participants were Dutch and performed the Dutch version of the task.^1^ The study was conducted in accordance with the principles expressed in the Declaration of Helsinki.

### Stimuli and materials

We selected 167 pictures of the Snodgrass and Vanderwart (1980) picture set to use as targets in the inducer task and as irrelevant cues in the visual probe task. In the instructions of the inducer task, we used the most often chosen object word to refer to the stimuli. All object words were presented in 24-point Courier New font. On visual probe trials, each object drawing was presented in black with a white background, inside a box that was 4.8 cm high and 6 cm wide (subtending 6.09 and 7.59 degrees of visual angle). These two boxes were presented 1.4 cm above and below fixation (subtending 1.78 degrees of visual angle). The fixation cross was presented in 16-point Courier New font. Targets in the visual probe task were the letter E and the letter F, presented in 18-point Courier New font. All stimuli, apart from the object drawings, were presented in white against a black background.

### Procedure

Participants were seated in a testing room with two or four desks, separated by partitions. We used an HP desktop PC with a 19-inch color LCD monitor. One, two, three or four participants could be tested at the same time. After giving informed consent, they performed the experiment. For stimulus presentation and response registration, we used the E-Prime software package (Schneider, Eschman, & Zuccolotto, 2002a, 2002b). Responses were recorded with a standard QZERTY keyboard.

The experiment consisted of nine blocks of 4 runs (two in which the inducer task question referred to the high-reward association, and two in which the inducer task question referred to the low-reward association), resulting in 36 runs (see Fig. 1 for a schematic example of a run). A run started with the presentation of the instructions for the inducer task. First, a prompt appeared that read “press the space bar for instructions”. Second, a screen appeared in which an object name (e.g., “BALCONY”) was presented together with the amount of points that was associated with the object (“+1”). This information remained on the screen for 4000 ms. Subsequently, a second instruction screen was presented with the second object name (e.g., “BOMB”), the other amount of points (“+10”). Thus, on each run, two objects were used, the first of which was related to low reward and the second was related to a high reward. Object names were picked randomly (without replacement) from the list of Snodgrass stimuli. After this, a screen appeared with the instruction to press the spacebar to begin.

This was followed by the presentation of the fixation cross for 1000 ms. Next, the two object pictures appeared each inside one of the boxes, for 500 ms. Then, the boxes were blank for 30 ms, after which the target (E or F) appeared either in the location of the high-reward stimulus (valid trial) or in the opposite location (invalid trial). Half of the trials were valid, and the other half were invalid. The target remained on screen until a response was given. Participants were instructed to respond as quickly and accurately as possible. After an incorrect response, the word “WRONG” appeared on the screen for 500 ms. A trial was followed by an inter-trial interval that lasted between 250 and 500 ms. During this interval, the fixation cross and the blank boxes remained present on the screen.

In line with previous research on the automatic effects of instructions (e.g., Liefooghe et al., 2012), half of the runs consisted of 8 visual probe trials; the other half consisted of 16 visual probe trials. This element of the procedure minimizes the anticipation of the presentation of the inducer probe and thus encourages them to always be ready to execute the instructions.

After the last visual probe trial of the run, one of the two inducer task questions appeared (i.e., “For which object do you earn 1 point?”; “For which object do you earn 10 points?”). After an incorrect response, the word “WRONG” was presented for 500 ms. If participants typed in a correct response, a screen appeared for 1000 ms with the feedback “CORRECT!” with below it the number of points they earned “+10” or “+1”, and below that “total points:” and the number of points they had earned so far. The run ended with a blank screen that was presented for a random duration lasting between 250 and 500 ms. Before the experiment, participants were told that participants who earned the most points would be eligible to enter a lottery for a €25 gift certificate. After completing the experiment, participants were thanked and asked to leave their email address if they wanted to be eligible for the lottery.

### Analyses

We analyzed the percentage of correct responses on the inducer task and compared high- and low-reward runs using paired-samples *t* tests. For the visual probe task, we labeled trials as “valid” when the probe appeared at the location of the high-reward stimulus and “invalid” when the probe appeared at the location of the low-reward stimulus. We compared the means on reaction times and accuracy for valid and invalid trials using paired-samples *t* tests. Finally, we performed Bayesian analyses on the relevant *t* tests to compare the fit of the data under the null hypothesis to the fit under the alternative hypothesis. We used the more conservative JSZ Bayes factor (Jarosz & Wiley, 2014; Rouder, Speckman, Sun & Morey, 2009).

## Results

We excluded data of 1 foreign participant who scored lower than 2.5 standard deviations below the mean accuracy on the inducer task and 2 participants (one Dutch, one foreign) whose reaction times were 2.5 standard deviations above the mean on the visual probe task, so the final sample consisted of 48 participants. We analyzed only the visual probe trials for runs on which the inducer task was performed correctly to be sure that participants had attended to and remembered the instructions of the inducer task.

### Inducer task

Participants performed well on the inducer task, with a mean accuracy rate of *M* = .94, SD = .06. There was no effect of reward value of the inducer stimulus *t* < .24. Overall, participants earned 187.08 points on average (SD = 12.87) out of a maximum of 198.

### Visual probe task

Our *t* test on the accuracy data reveal no significant differences between valid, *M* = .97, SD = .02, and invalid trials, *M* = .97, SD = .02, *t* < .82. However, our reaction time analyses show that the difference between trial types was highly significant, *t*(47) = 3.77, *p* < .001, *d* = .15. Participants were faster on valid, *M* = 639, SD = 75, 95% CI [628, 650], compared to invalid trials, *M* = 651, SD = 80, 95% CI [640, 663]. The BF10 was 58.49, suggesting there is strong evidence that responses were faster for valid compared to invalid trials (Jefferies, 1961).

## General discussion

Our study is the first to show that attention is biased toward stimuli that are paired with high relative to low reward on the basis of instructions. The fact that these effects had not yet been examined is likely due to the prominent assumption, which suggests that automatic attentional bias for rewarding stimuli is the result of training (e.g., Pessoa, 2014). This view suggests that the repeated presentation of a neutral CS with a rewarding US leads to the formation of CS–US associations in memory, which results in a transfer of the salience of the US to the CS (e.g., Anderson, 2016; Awh et al., 2012; Theeuwes, 2018). Our findings cannot be explained by these models: participants’ expectations regarding future stimulus–reward pairings are sufficient to bias their attention toward these stimuli even when they have no prior experience with the objects and no prior experience with the contingencies between the objects and the rewards at the beginning and during the each run. Importantly, the objects were not visually salient (i.e., there was no effect of bottom-up processes). Furthermore, it was not beneficial to attend to objects presented in the visual probe task (i.e., there was no effect of top-down processes^2^) for two reasons. First, the physical properties of the objects were not relevant (i.e., participants only needed to remember the associations between words and points, whereas the stimuli in the visual probe task were pictures). Second, participants could only be entered in the lottery for the gift certificate if they earned the most points. Thus, both high- and low-reward stimuli were equally important to be eligible for the lottery, and it was not beneficial to preferentially attend to or rehearse the high-reward stimuli relative to the low-reward stimuli. Instead, our data support novel learning theories that have so far been overlooked within the literature regarding reward effects on attention. These models propose that prior knowledge, instructions, expectations, and deductive reasoning can result in similar effects as actual CS–US pairings (e.g., De Houwer, 2009; Gawronski & Bodenhausen, 2011; Ramamoorthy & Verguts, 2012).

We believe that these theories open promising avenues for future research and generate novel questions regarding attentional and other effects of rewards. For instance, it is crucial to examine mechanisms related to and possibly underlying the attentional bias toward instructed high-reward compared to instructed low-reward stimuli. Whereas experienced reward has widely been reported to affect visual attention (e.g., Anderson, 2016), it has also been reported to affect a range of other cognitive processes such as visual perception (e.g., Marx & Einhäuser, 2015; Wilbertz, Van Slooten, & Sterzer, 2014), visual working memory (e.g., Infanti, Hickey, & Turatto, 2015; Wallis, Stokes, Arnold, & Nobre, 2015), and conflict adaptation (Krebs, Boehler, & Woldorff, 2010). Furthermore, future studies need to examine the extent to which effects of instructed stimulus–reward pairings differ from the effects of trained stimulus–reward pairings.

Our study also allows us to shed light on dissociations between selection and reward. According to Awh et al. (2012) there is a distinction between attentional control on the basis of selection history and attentional control on the basis of reward history. However, in studies concerning effects of reward learning on attention, these two histories are confounded: in a typical experiment, participants learn to selectively attend to a reward-related stimulus during a training phase. In a subsequent test phase, their attention is biased toward this stimulus (e.g., Anderson et al., 2012). Note that this can be due both to their selection history (i.e., they are extensively trained to attend to this specific stimulus and to ignore other stimuli) as well as to their reward history (i.e., they received ample experience of the stimulus–reward pairing). Our study is not only the first to reveal the automatic effects of reward when there are not stimulus–reward pairings, but it is also the first study to tease apart selection and reward effects.

As mentioned in our introduction, we previously found automatic effects of future action selection (i.e., instructed S-R mappings; Tibboel et al., 2016). However, in the same study we also performed an experiment in which we examined the effects of instructed S–S mappings instead of instructed S–R mappings on attention. In this case, the instructions for the inducer task stated that a particular stimulus (e.g., a picture of a balcony) was paired with another stimulus (i.e., the color green) and that participants needed to remember this pairing. At the end of the run, a picture would appear in either the specified color (e.g., a green balcony) or another color (e.g., a blue balcony) and participants needed to indicate whether the pairing was in line with the instructions. In this experiment, stimuli were thus not associated with a particular response (i.e., there was no S–R mapping), but only with a particular stimulus (i.e., an S–S mapping). Results showed that these future S–S mappings failed to bias attention. In the current study, however, the attentional bias can be due only to the instructed stimulus–reward mapping (an S–S mapping) and not due to instructed S-R mappings, as both stimuli were associated with a response. In other words, our studies suggest that both future selection and future reward have separate effects on attentional processing.

Our findings do not only have important theoretical implications, but they are also relevant for contexts in which reward processing is maladaptive. Maladaptive reinforcement learning is assumed to play an important role in addiction and schizophrenia (e.g., Frank, 2008; Robinson & Berridge, 2003) and cognitive bias modification (CBM) procedures have been developed to change these biases on the basis of direct experience (e.g., Cox). However, questions have risen regarding the processes underlying CBM effects (e.g., Beard, 2011; Heeren et al., 2013; Van Bockstaele et al., 2014). A better understanding of how automatic bias toward reward-related stimuli develop (e.g., through direct experience or through derivation) can eventually steer us toward better interventions to change these biases.

Finally, we must note that we find a relatively strong attentional bias toward high- relative to low-reward stimuli, and the question remains how this effect holds up to effects of experience. Automatic effects on the basis of instructions are often smaller than automatic effects on the basis of experience (e.g., Van Dessel et al., 2015; Wenke et al., 2015). Even though we did not directly compare experienced and instructed stimulus–reward pairings, our effect sizes are smaller than those reported in reactive training studies (e.g., Anderson et al., 2011). Our data support accounts suggesting that experience might not be crucial to obtain automatic effects and experience does further consolidate the instructed contingencies (e.g., Wenke et al., 2015).

## Footnotes

1 We analyzed the data with language as a between subject factor, this did not affect the data pattern.

2 It is important to note that there is still a debate about the definition of bottom-up and top-down processing. Whereas Theeuwes (2018) defines top-down attentional control as voluntary or goal-driven, others consider any effects of context or expectations a form of top-down control (e.g., Gaspelin & Luck, 2018). Importantly, even with this broader definition, our study contributes to the reward literature by showing that expectations regarding stimulus–reward contingencies affect automatic attention in an unrelated task.
